# Real-world efficacy of direct acting antiviral therapies in patients with HIV/HCV

**DOI:** 10.1371/journal.pone.0228847

**Published:** 2020-02-13

**Authors:** Sonia Vibhakar Patel, Dushyantha T. Jayaweera, Keri N. Althoff, Joseph J. Eron, Janna Radtchenko, Anthony Mills, Graeme Moyle, Steven Santiago, Paul E. Sax, Jason Gillman, Karam Mounzer, Richard A. Elion, Gregory D. Huhn

**Affiliations:** 1 The Ruth M. Rothstein Core Center, Cook County Health, Chicago, Illinois, United States of America; 2 Division of Infectious Disease, Miller School of Medicine, University of Miami, Miami, Florida, United States of America; 3 Johns Hopkins University, Baltimore, Maryland, United States of America; 4 Division of Infectious Diseases, University of North Carolina at Chapel Hill, Chapel Hill, North Carolina, United States of America; 5 Trio Health Analytics, La Jolla, California, United States of America; 6 Southern California Men’s Medical Group, Los Angeles, California, United States of America; 7 Chelsea and Westminster Hospital, London, United Kingdom; 8 Care Resource, Miami, Florida, United States of America; 9 Brigham and Women’s Hospital and Harvard Medical School, Boston, Massachusetts, United States of America; 10 Prism Health North Texas, Dallas, Texas, United States of America; 11 Perelman School of Medicine at The University of Pennsylvania and Philadelphia Fight Community Health Centers, Philadelphia, Pennsylvania, United States of America; 12 George Washington University School of Medicine, Washington, District of Columbia, United States of America; 13 The Ruth M. Rothstein Core Center and Rush University Medical Center, Chicago, Illinois, United States of America; National Taiwan University Hospital, TAIWAN

## Abstract

The advent of direct-acting antiviral (DAA) therapies has dramatically transformed HCV treatment, with most recent trials demonstrating high efficacy rates (>90%) across all genotypes and special populations, including patients with HIV/HCV coinfection. The efficacy rates of HCV treatment are nearly identical between patients with HCV monofection and patients with HIV/HCV coinfection; however, there are limited studies to compare real-world efficacy with efficacy observed in clinical trials. Using a database from HIV clinics across the United States (US), we identified 432 patients with HIV/HCV coinfection who completed DAA therapy from January 1, 2014 to March 31, 2017 and were assessed for efficacy. Efficacy was evaluated as sustained virologic response (SVR) 12 weeks after DAA completion; furthermore, factors associated with achieving SVR12 were identified. In this analysis, we found DAA therapies to be effective, with 94% of the patients achieving SVR12 and 6% experiencing virologic failure. Baseline variables, including older age, HCV viral load <800K IU/ML, FIB-4 score <1.45, absence of depression, diabetes, substance abuse, and use of DAA regimens without ribavirin were significant predictors of achieving SVR12. Patients with fewer comorbidities, better liver health, and lower HCV viral loads at baseline were more likely to achieve treatment success. Our results were consistent with other real-world studies, supporting the use of HCV therapy in HIV/HCV coinfected patients.

## Introduction

It is estimated that 2.27 to 2.75 million people around the world have hepatitis C virus (HCV) and human immunodeficiency virus (HIV) coinfection.[1–3] In the United States (US), of the people living with HIV (PLWH), about 25% are estimated to have HCV infection, which consequently triples the risk for liver disease, liver failure, and liver related death.[4] Nearly 75% to 82% of PLWH who inject drugs are also infected with HCV.[2,4,5] Prior to potent combination antiretroviral therapy (ART), early studies suggested that progression to fibrosis is accelerated in patients with HIV/HCV coinfection. Factors affecting disease progression in this population include increased alcohol use, older age, longer duration of HCV infection, and immunosuppression (CD4 < 200 cells/mm^3^). More recent data, however, show a similar course of progression in HIV positive patients primarily due to sufficient immune reconstitution in the modern ART era.[6–9] Notwithstanding, HIV/HCV coinfection has been associated with increased risk of death in large cohort studies compared to HIV monoinfection.[9–12] Advanced liver disease accounted for 13% of all deaths among PLWH prior to treatment with direct-acting antivirals (DAAs), and in the US chronic HCV infection has been shown to be the leading cause of liver disease and related mortality in coinfected individuals.[13–17]

In the past, persons with HIV/HCV coinfection achieved lower rates of sustained virologic response (SVR) after use of interferon and ribavirin HCV treatment compared with those with HCV mono-infection.[5] In addition to poor efficacy, older HCV treatment regimens were associated with many adverse effects such as depression, anemia, flu-like symptoms, and were often contraindicated due to additional comorbidities within the PLWH.[18–20] With the development of interferon-free DAA therapies, the treatment platform for PLWH has been revolutionized and efficacy in patients with HIV/HCV coinfection has improved. There are recent published clinical trials that reported high rates of SVR (>90%) with DAA therapy in patients with HIV/HCV coinfection.[21] However, the rigid inclusion criteria in these clinical trials may not be an accurate representation of patients with HIV/HCV coinfection in clinical care. [22–27] Many co-infected patients, especially those in urban clinics, struggle with psychosocial issues such as drug and alcohol abuse, unstable housing, and mental health disparities. Access to medications due to individual state restrictions as well as drug interactions with antiretrovirals remain a challenge in this patient population. Substance abuse remains a common restriction on many state Medicaid plans.[20, 28–31]

HCV treatment can be complicated with the number of drug-drug interactions associated with antiretroviral therapies; however, the Department of Health and Human Services (DHHS) recommends first-line ART regimens with minimal drug interactions and that are compatible with most DAA therapies. There has been concern that SVR results obtained in clinical trials may not be generalizable due to the selection of patients or stringent inclusion criteria; however a number of real-world studies in the US have been completed and reported high rates of both adherence and SVR in the HIV/HCV coinfected population.[32–38] Some of these studies have evaluated efficacy of specific DAA therapies in one clinic/hospital setting with small patient sample sizes (range 30–109 patients) using US data.[32,33,35,37,38] One study by Del Bello et al evaluated predictors of treatment failure, which included fibrosis-4 (FIB-4) score ≥3.25, low AST levels, and use of simeprevir/sofosbuvir/ribavirin (SMV/SOF/RBV); however, the sample consisted of 89 patients with genotype 1 treated in a hospital setting in New York, USA.[34] These studies have added valuable real-world evidence by reporting high SVR rates comparable to those seen in clinical trials, but had some limitations such as small patient numbers, use of data prior to 2018, and single sites and thus are not easily generalizable to other institutions and geographic regions.

There are limited data from multi-site HIV clinics across the US, including patients with multiple HCV genotypes and all DAA therapies, and identifying factors associated with treatment response. Our objective for this analysis was to evaluate the SVR rates assessed at 12 weeks after completion of DAA therapy and to identify predictors of SVR12 achievement among patients with HIV/HCV coinfection using a multi-site HIV database.

## Materials and methods

### Study design and data

This was an observational retrospective cohort study using real-world data with linked electronic medical records (EMR) and pharmacy dispense data from a proprietary longitudinal database established by Trio Health. The database contains records of over 38,000 HIV patients from 50 US states and Puerto Rico. The data generated through the EMR are available for research purposes and include information on patient clinical and demographic characteristics, prescriptions and dispenses, coded diagnoses, and laboratory test results collected at 11 large HIV-treating US clinics since 2014. This study was approved as an Institutional Review Board (IRB) exemption under category 45 CFR 46 that waived the requirement for informed consent, since this was a retrospective database study without direct patient contact using deidentified data.

### Study population

Patients coinfected with HCV/HIV, aged ≥18 years old, who initiated DAA +/- ribavirin therapies (HCV regimen) from January 1, 2014 to March 31, 2017 were identified. The index date was defined as the date of HCV regimen initiation using pharmacy dispensing records. Patients were followed until their SVR was assessed at 12 weeks after completion of their HCV regimen. Patients were further categorized as achieving SVR12 and not achieving SVR12. Baseline characteristics included, age, gender, ethnicity, geographic region, body mass index (BMI), fibrosis burden (FIB-4 score), transplant status, and comorbidities (cancer, cirrhosis, depression, diabetes, hepatitis B infection, heart disorders [cardiovascular disease, coronary artery disease, aortic aneurysm], chronic kidney disease, substance abuse). Collected laboratory values included HCV genotype, initial viral load, alanine aminotransferase (ALT) levels, aspartate aminotransferase (AST) levels, total bilirubin levels, absolute CD4+ T-cell counts (CD4 count), and HIV viral load.

### Study outcomes

The outcomes of the study were to evaluate the rates of SVR12 and to identify factors associated with SVR12 among HCV/HIV coinfected patients. SVR12 was defined as an undetectable plasma HCV RNA at 12 weeks after completion of HCV regimen.

### Statistical analysis

Unadjusted descriptive statistics summarized patient characteristics of the two study cohorts (achieving SVR12 versus not achieving SVR12). Differences between these patient groups were tested using Student’s *t* test or Mann-Whitney test for continuous variables and chi squared statistic or Fisher’s exact tests for categorical variables. A multivariable logistic regression model was generated with SVR12 success as the dependent variable. Some of the potential risk factors for the model included demographic information (age, gender, ethnicity, geographic region); indices of liver disease severity (cirrhosis, FIB-4 score>3.25, and liver transplant); HCV genotype; laboratory results such as HCV viral load, absolute CD4 cell count, ALT, AST levels; comorbidities (depression, diabetes, chronic kidney disease, substance abuse, etc.). A backwards stepwise selection process was used to include factors with the significance level of *P* ≤ 0.2 for the final model. Odds ratio (OR) and 95% confidence intervals (95% CI) for predictors of achieving SVR from the logistic regression model were reported. All analyses were conducted using SAS 9.4 software (SAS Institute, Cary, NC). A two-sided P value <0.05 was considered statistically significant.

## Results

**Fig 1:**
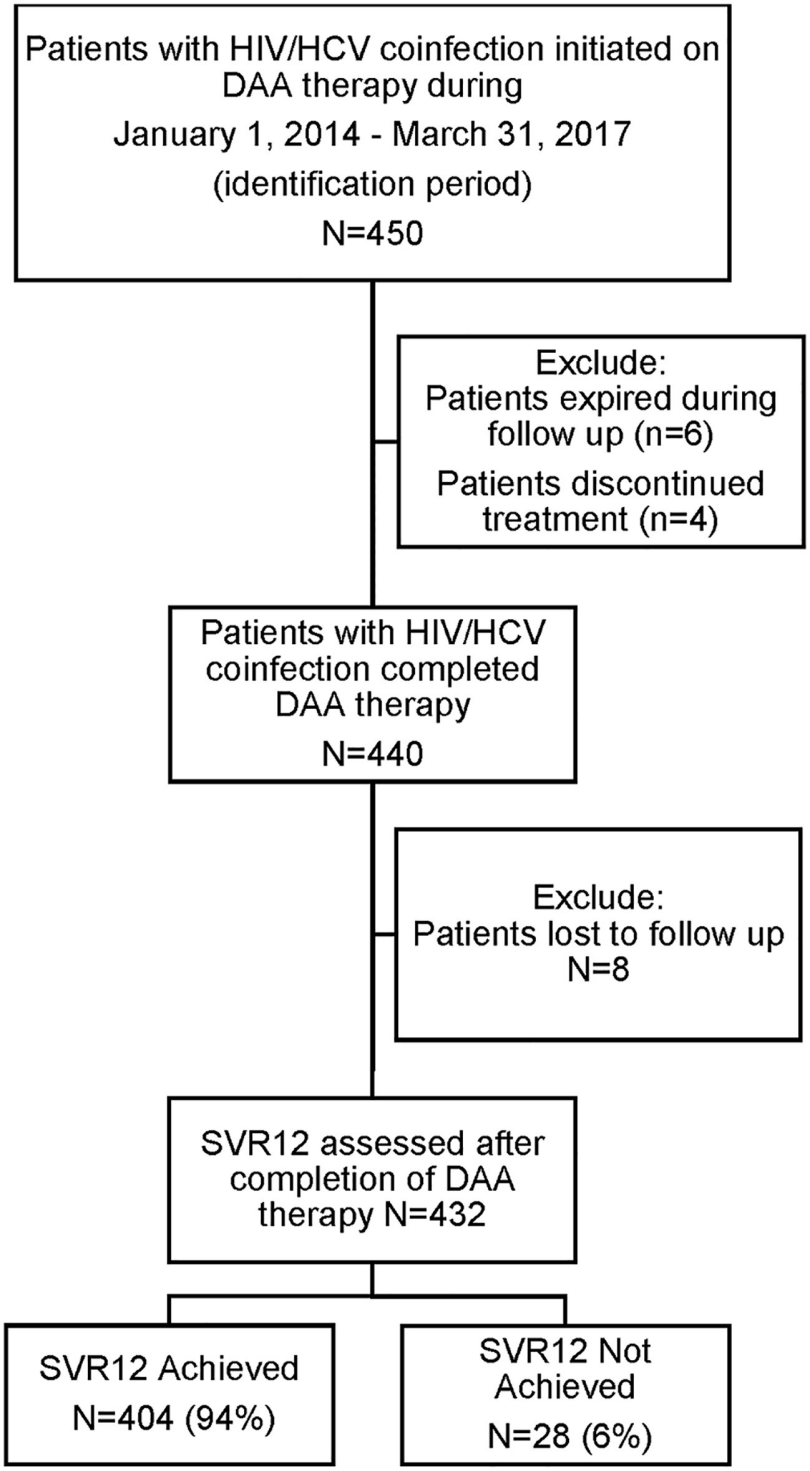
Patient cohort flow diagram. DAA = direct acting antiviral; HIV = human immunodeficiency virus; HCV = hepatitis C virus; SVR12 = sustained virologic response assessed 12 weeks after completion of DAA therapy.

A total of 450 HCV/HIV coinfected patients were identified on HCV regimens from January 1, 2014 to March 31, 2017. Of them, 6 patients died during the follow-up period, 4 discontinued treatment, and 8 were lost to follow-up. The final study cohort included 432 patients who completed DAA therapy and were evaluated for SVR12 (Fig 1).

Of the patients who completed therapy and were assessed for SVR12, 404 patients (94%) achieved SVR12 and 28 patients (6%) experienced virologic failure.

### Patient characteristics

The baseline clinical and demographic patient characteristics are described in Table 1. Patients who achieved SVR12 (SVR12 group) were older than those who did not achieve SVR12 (non-SVR12 group) with mean age 55.3 years vs 51.7 years as shown in Table 1.

**Table 1:** Baseline clinical and demographic characteristics.

	Total Population N = 432	SVR12 N = 404	No SVR12 N = 28	P-value^a^
Age (years), mean ± SD	55.7±9.7	55.3±9.2	51.7±9.4	**0.039**
**Gender, n (%)**				0.561
Male	335 (77.5%)	312 (77.2%)	23 (82.1%)	
Female	96 (22.2%)	91 (22.5%)	5 (17.8%)	
Unknown	1 (0.2%)	1 (0.3%)	0 (0.0%)	
**Ethnicity, n (%)**				0.101
Black	236 (54.6%)	225 (55.7%)	11 (39.3%)	
White	101 (23.4%)	90 (22.3%)	11 (39.3%)	
Hispanic	73 (16.9%)	67 (16.6%)	6 (21.4%)	
Unknown	22 (5.1%)	22 (5.5%)	0 (0.0%)	
**Insurance Type, n (%)**				0.587
Commercial	54 (12.5%)	51 (12.6%)	3 (10.7%)	
Medicaid	148 (34.3%)	136 (33.7%)	12 (42.9%)	
Medicare	81 (18.7%)	78 (19.3%)	3 (10.7%)	
Self-pay	13 (3.0%)	13 (3.2%)	0 (0.0%)	
Other or unknown	136 (31.4%)	126 (31.2%)	10 (35.7%)	
**Geographic Region, n (%)**				0.819
Northeast	193 (44.7%)	179 (44.3%)	14 (50.0%)	
Midwest	150 (34.7%)	142 (35.2%)	8 (28.6%)	
South	84 (19.4%)	78 (19.3%)	6 (21.4%)	
West	5 (1.2%)	5 (1.2%)	0 (0.0%)	
**BMI**^b^**, n (%)**				0.893
Underweight: <18.5	8 (1.8%)	8 (2.4%)	0 (0.0%)	
Normal weight: 18.5 to 24.9	168 (28.9%)	156 (46.0%)	12 (48.0%)	
Overweight: > 25.0 to 29.9	103 (23.8%)	96 (28.3%)	7 (28.0%)	
Obese: ≥30	85 (19.7%)	79 (23.3%)	6 (24.0%)	
Unknown	68 (15.7%)	65 (16.1%)	3 (10.7%)	
**Genotype, n (%)**				
Not genotyped	2 (0.5%)	2 (0.5%)	0 (0.0%)	0.713
Genotyped				
GT1A^c^	275 (63.7%)	256 (63.4%)	19 (67.9%)	0.936
GT1B	92 (21.2%)	87 (21.5%)	5 (17.9%)	
GT1X	7 (1.6%)	6 (1.5%)	1 (3.6%)	
GT2	25 (5.8%)	23 (5.7%)	2 (7.1%)	
GT3	27 (6.3%)	26 (6.4%)	1 (3.6%)	
GT4	4 (0.9%)	4 (1.0%)	0 (0.0%)	
**Baseline Labs**				
CD4 count, n (%)	325 (75.2%)	303 (75.0%)	22 (78.6%)	0.826
CD4 count <200 cells/ mm^3^, n (%)	27 (6.3%)	24 (7.9%)	3 (13.6%)	**0.035**
Initial HCV VL^d^, n (%)	358 (82.9%)	334 (82.7%)	24 (85.7%)	0.128
HCV VL <800K IU/ML	92 (21.3%)	90 (27.0%)	2 (8.3%)	**0.021**
HCV VL 800K-6MM IU/ML	210 (48.6%)	193 (57.8%)	17 (70.8%)	
HCV VL >6MM IU/ML	56 (12.9%)	51 (15.3%)	5 (20.8%)	
HIV VL, n (%)	357 (82.6%)	334 (82.7%)	23 (82.1%)	0.701
HIV VL <200 copies/ML, n (%)	336 (77.8%)	315 (94.3%)	21 (91.3%)	0.553
ALT^e^, n (%)	349 (80.8%)	324 (80.2%)	25 (89.2%)	0.611
ALT (IU/L), mean ± SD	62.1±62.8	59.5±59.2	83.9±77.6	**0.012**
ALT >55 IU/L, n (%)	118 (27.3%)	106 (26.2%)	12(42.9%)	**0.001**
AST^f^, n (%)	342 (79.2%)	319 (78.9%)	23 (82.1%)	0.419
AST (IU/L), mean ± SD	60.5±53.4	54.9±37.0	89.5±78.9	**0.019**
AST >48 IU/L, n (%)	144 (33.3%)	129 (31.9%)	15 (53.6%)	**0.037**
Total Bilirubin, n (%)	349 (80.8%)	324 (80.2%)	25 (89.2%)	0.621
Total Bilirubin (mg/DL), mean ± SD	0.9±1.1	0.7±0.6	1.1±1.4	0.512
Total Bilirubin >1.2 mg/DL, n (%)	45 (10.4%)	40 (9.9%)	5 (17.8%)	**0.022**
FIB-4^g^ n, (%)	334 (11.1%)	312 (77.2%)	22 (78.5%)	0.712
FIB-4 < 1.45	92 (21.3%)	89 (28.5%)	3 (13.6%)	**0.012**
FIB-4 1.45to ≤ 3.25	161 (37.3%)	151 (48.4%)	10 (45.5%)	
FIB-4 > 3.25	81 (18.8%)	72 (23.1%)	9 (40.9%)	
**Comorbidities, n (%)**				
Asthma	62 (14.4%)	59 (14.6%)	3 (10.7%)	0.570
Cancer	32 (7.4%)	28 (6.9%)	4 (14.3%)	**0.016**
Cirrhosis	100 (23.1%)	89 (22.0%)	11 (40.7%)	**0.026**
Depression	148 (34.3%)	134 (33.1%)	14 (50.0%)	**0.039**
Diabetes	59 (13.6%)	53 (13.1%)	6 (21.4%)	**0.022**
Heart Disorders^h^	68 (15.7%)	67 (16.6%)	1 (3.6%)	**0.016**
Hepatitis B Infection	11 (2.5%)	9 (2.2%)	2 (7.1%)	**0.031**
Hypertension	186 (43.1%)	173 (42.8%)	13 (46.4%)	0.709
Hyperlipidemia	76 (17.6%)	72 (17.8%)	4 (14.3%)	0.635
Chronic kidney disease	53 (12.2%)	48 (11.9%)	5 (17.9%)	**0.035**
Liver transplant recipient	1 (0.2%)	0 (0.0%)	1 (3.6%)	0.065
Psychiatric Disorders	87 (20.1%)	81 (20.1%)	6 (21.4%)	0.860
Substance abuse	157 (36.3%)	141 (34.9%)	16 (57.1%)	**0.018**
Tobacco use	199 (46.1%)	183 (45.3%)	16 (57.1%)	**0.022**
**DAA Therapies**				**0.001**
+ RBV^i^, n (%)	70 (16.2%)	59 (14.6%)	11 (39.3%)	
- RBV, n (%)	362 (83.8%)	345 (85.4%	17 (60.7%)	

^a^ P-value <0.05 was considered statistically significant.

^b^ BMI = body mass index.

^c^ GT = genotype.

^d^ VL = viral load.

^e^ ALT = alanine aminotransferase.

^f^ AST = aspartate aminotransferase.

^g^ FIB-4 = fibrosis-4 score for liver.

^h^ Heart disorders = cardiovascular disease, coronary artery disease, aortic aneurysm.

^i^ RBV = ribavirin.

There were no statistically significant differences between the two study groups by gender, ethnicity, insurance type, geographic region, BMI categories, or HCV genotype. Most of the patients (>70%) had genotype 1 infection (GT1A, GT1B, and GT1X) within both groups. Compared to the SVR12 group, the non-SVR12 group had significantly higher proportion of patients with CD4 counts <200 cells/mm^3^, HCV viral load >800K IU/ML, and FIB-4 score >3.25 as well as higher mean ALT, AST, and total bilirubin. The SVR12 group had fewer comorbidities than the non-SVR12 group, with lower rates of cancer (6.9% vs 14.3%, p = 0.016), cirrhosis (22.0% vs 40.7%, p = 0.026), depression (33.1% vs 50.0%, p = 0.039), diabetes (13.1% vs 21.4%, p = 0.022), hepatitis B infection (2.2 vs 7.1%, p = 0.031), chronic kidney disease (11.9 vs 17.9%, p = 0.035), substance abuse (34.9% vs 57.1%, p = 0.018), and tobacco use (45.3% vs 57.1%, p = 0.022).

### HCV regimens

Patients in the SVR12 group were mostly on DAA therapies without concomitant ribavirin (RBV) therapy compared to the non-SVR12 group (85.4% [n = 345] vs 60.7% [n = 17]). The majority of SVR12 patients (67.3% [n = 272]) received ledipasvir-sofosbuvir (LDV+SOF) compared to 46.4% (n = 13) of the patients in the non-SVR12 group. Patients in the non-SVR12 group were more likely to be on DAA with concomitant RBV therapy (39.3% [n = 11]) compared to SVR12 group (14.6% [n = 59]). Patients who did not receive ribavirin therapy achieved treatment success 95.3% of the time, while those who received ribavirin achieved SVR12 84.3% of the time. Table 2 lists treatment success rates by regimen.

**Table 2:** HCV therapies and treatment success.

	Total Population N = 432	SVR12 N = 404	No SVR12 N = 28
HCV Therapies	N (%)	n (% SVR12)	n (% No SVR12)
**DAA +RBV**^a^	70 (16.2%)	59 (84.3%)	11 (15.7%)
SOF^b^ + RBV	39 (9.0%)	32 (82.1%)	7 (17.9%)
PROD^c^ + RBV	13 (3.0%)	10 (76.9%)	3 (23.1%)
SOF + PEG^d^ + RBV	6 (1.4%)	6 (100.0%)	0 (0.0%)
LDV^e^-SOF + RBV	5 (1.2%)	5 (100.0%)	0 (0.0%)
SMV^f^ + SOF + RBV	4 (0.9%)	3 (75.0%)	1 (25.0%)
SOF-VEL^g^ + RBV	2 (0.5%)	2 (100.0%)	0 (0.0%)
DCV^h^ + SOF + RBV	1 (0.2%)	1 (100.0%)	0 (0.0%)
**DAA—RBV**	362 (83.8%)	345 (95.3%)	17 (4.7%)
LDV-SOF	285 (66.0%)	272 (95.4%)	13 (4.6%)
SMV + SOF	29 (6.7%)	27 (93.1%)	2 (6.9%)
EBR-GZR^i^	17 (3.9%)	16 (94.1%)	1 (5.9%)
DCV + SOF	15 (3.5%)	15 (100.0%)	0 (0.0%)
SOF + VEL	11 (2.5%)	10 (90.9%)	1 (9.1%)
PROD	5 (1.2%)	5 (100.0%)	0 (0.0%)

^a^ RBV = ribavirin.

^b^ SOF = sofosbuvir.

^c^ PROD = paritaprevir/ritonavir-ombitasvir and dasabuvir.

^d^ PEG = peginterferon.

^e^ LDV = ledipasvir.

^f^ SMV = simeprevir.

^g^ VEL = velpatasvir.

^h^ DCV = daclatasvir.

^i^ EBR-GZR = elbasvir-grazoprevir.

### Predictive factors associated with SVR12 success

In the final multivariable logistic regression model, several factors were associated with achieving SVR12 Table 3.

**Table 3:** Factors associated with SVR12 achievement.

Patient Characteristics	Odds Ratio (95% CI)	P-value^a^
Age	1.14 (1.03–1.27)	**0.011**
Cirrhosis	0.34 (0.09–1.34)	0.126
Depression	0.82 (0.02–0.96)	**0.040**
Diabetes	0.68 (0.13–0.94)	**0.028**
Substance abuse	0.48 (0.07–0.97)	**0.039**
**Baseline Initial Viral Load**		
HCV VL^b^ <800K IU/ML	13.77 (5.37–18.82)	**0.026**
HCV VL 800K to 6MM IU/ML	1.00	-
HCV VL >6MM IU/ML	0.57 (0.16–2.08)	0.398
**Baseline FIB-4**^c^		
FIB-4 <1.45	5.18 (1.12–8.28)	**0.008**
FIB-4 1.45 to ≤3.25	1.00	-
FIB-4 >3.25	0.54 (0.16–1.84)	0.327
**Baseline CD4 count**		
≥200 cells/mm^3^	1.00	-
<200 cells/mm^3^	0.42 (0.11–1.92)	0.265
**DAA Therapies**		
HCV regimen—RBV^d^	1.00	-
HCV regimen + RBV	0.13 (0.03–0.60)	**0.009**

^a^ C-statistic (model fit) = 0.899; p-value <0.05 was considered statistically significant.

^b^ VL = viral load.

^c^ FIB-4 = fibrosis-4 score for liver.

^d^ RBV = ribavirin.

Older patients were more likely to achieve SVR12 (OR 1.14, 95 CI: 1.03–1.27, p = 0.011). Patients with baseline depression (OR 0.82, 95 CI: 0.02–0.96, p = 0.040), diabetes (OR 0.68, 95 CI: 0.13–0.94, p = 0.028), substance abuse (OR 0.48, 95 CI: 0.07–0.97, p = 0.039), and on ribavirin therapy (OR 0.13, CI: 0.03–0.60, p = 0.009) were less likely to achieve SVR12. Baseline HCV viral load <800K IU/ML was associated with 13 times greater likelihood of achieving SVR12 versus having an HCV viral load between 800K to 6MM IU/ML (OR 13.77, CI: 5.37–18.82, p = 0.026). Patients with FIB-4 score <1.45 were 5 times more likely to achieve SVR12 (OR 5.18, 95% CI: 1.12–8.28, p = 0.008) compared to those with FIB-4 1.45 to ≤3.25.

## Discussion

To our knowledge, this is one of the largest US cohort studies of patients with HIV/HCV coinfection who initiated DAA therapies in a real-world setting. In this analysis, we observed SVR12 rates of 94%, consistent with prior real-world studies showing >90% SVR rates. [32,35,38] Current guidelines[39] recommend that patients with HIV/HCV coinfection be treated using the approach followed for non-HIV infected patients, because efficacy of current DAA regimens does not differ between HCV-mono-infected and coinfected patients, while PLWH represent an especially vulnerable population. In HIV-infected men having sex with men (MSM) HCV transmission continues to rise; therefore, HCV elimination efforts directed toward this population will be important to decrease the HCV reservoir to prevent further transmission. [40–42] Treatment of HCV as prevention in this high risk population is critical to decrease the overall HCV incidence in PLWH, particularly with the expansion of the HIV U = U (undetectable = untransmissible) message in clinics across the US.[43,44] Although an empowering message for PLWH, the U = U message has the potential to increase the incidence of acute HCV in patients engaging in condomless sex.[45,46]

Possible barriers to a successful HCV response in PLWH include access to medical care, timely diagnosis of HIV/HCV and initiation of DAA therapy, costs associated with therapy, underinsured status, longer time needed for screening HIV/HCV and time required for counseling.[5,47] Achieving SVR12 has also been shown to affect quality of life (QoL) in patients with HIV/HCV coinfection. Prior to DAA therapy availability, QoL reported by patients with HIV/HCV coinfection was significantly lower than in HIV mono-infected patients.[16,48] However, after completion of DAA therapy, QoL scores were comparable between the two study groups, and DAA therapy demonstrated improvement in general health and emotional well-being of patients with HIV/HCV coinfection.[48] Overall, the advent of DAA therapy has had a positive impact on this population.

High SVR rates achieved with DAA therapy and the small number of patients included in the registration trials have made it difficult to identify factors associated with treatment failure in both monoinfected and coinfected patients.[49] Therefore, we evaluated predictors of treatment success. Based on baseline characteristics, patients with SVR12 had lower rates of comorbidities, lower HCV viral loads and ALT, AST levels, and FIB-4 score compared to the non-SVR group. The descriptive findings were consistent with the multivariable model, where patients with older age, less fibrotic liver (FIB-4 score <1.45) and lower levels of HCV initial viral load (<800K IU/ML) were more likely to achieve treatment success (SVR12). Factors such as depression, diabetes, substance abuse, HCV viral load >6MM IU/ML, FIB-4 score >3.25, CD4 count <200 cells/mm^3^, and DAA plus ribavirin therapy were independently associated with not achieving SVR12. However, these findings should not be interpreted as lack of DAA efficacy in these groups as SVR12 rates were still high in patients with CD4 <200 cells/mm^3^ (88.8% [n = 27]), depression (90.5% [n = 148]), and substance abuse (89.8% [n = 157]).

Prior studies supported our findings as related to factors associated with not achieving SVR12. Berenguer et al found male sex, CD4 count <200 cells/mm^3^, HCV RNA viral load ≥800K IU/mL, compensated cirrhosis, and the use of sofosbuvir plus ribavirin were associated with treatment failure based on the analysis of patients in Madrid, Spain.[49] Del Bello et al found that patients receiving simeprevir/sofosbuvir/ribavirin therapy were more likely to have treatment failure.[34] Tapper et al found that presence of cirrhosis, prior treatment experience, treatment at an academic center, and treatment outside of the FDA recommendations were each associated with lower odds of achieving SVR.[50]

In the few patients who experienced treatment failure, active drug and alcohol use and depression were the greatest predictors of not achieving SVR. Based on prior studies, drug use within 6 months of initiating HCV therapy is not associated with decline in response to treatment; however, more frequent drug use correlates with decreased efficacy of treatment.[51–55] Social functioning, including unstable housing, transportation difficulties, stigmatization, and clinic attendance are better indicators of treatment outcome, independently associated with SVR after adjusting for drug use.[56] International guidelines support prioritizing persons at risk for transmitting HCV, including active injection drug users, to reduce the duration that the person is infectious.[57] Colocation of HIV care, HCV testing and treatment, medically assisted treatment for illicit drug use, and mental health services, as offered in the majority of the clinical settings in our study, can increase success rates on HCV therapy in this population.[58,59]

Limitations of this multicenter retrospective study include lack of uniformity of staging modalities other than FIB-4 calculations to determine severity of fibrosis, which may have generated variability in center-specific HCV treatment decisions. In addition, many coinfected patients with active drug or alcohol abuse within these HIV centers may have been excluded from DAA eligibility due to strict individual state Medicaid restrictions denying access to these medications, which may limit the generalizability of these results to these subpopulations.[60] In the Canadian Coinfection Cohort (CCC), a representative HIV/HCV population in an industrialized country was used to evaluate the percentage of current cohort participants who would be eligible to participate in all oral DAA clinical trials. Exclusivity to specific antiretroviral therapies as well as illicit drug use excluded greater than 50% of the HIV co-infected population from receiving HCV treatment.[61] Individual state level Medicaid restrictions in several jurisdictions in the US also require rigid criteria as to who has access to DAA therapy, potentially limiting these curative therapies among PLWH.[30]

Additionally, due to the heterogeneity of HIV regimens in the sample, they were not included in the model. Lastly, the database platform does not record DAA adherence patterns, a behavioral factor central to successful DAA treatment. However, shorter courses of treatment and the inclusion of the dispensing records in this database provides some reassurance that the medications were taken properly.

## Conclusions

Currently, there are limited real-world data on HCV treatment outcomes for patients with HIV/HCV coinfection. This large, real-world, multi-site, US study evaluating rates of SVR12 achievement and factors associated with treatment success significantly contributes to evidence in the coinfected population. In this analysis of patients with HIV/HCV coinfection, we found DAA therapies to be effective, with 94% of the patients achieving SVR12, consistent with rates seen in monoinfected HCV patients. Baseline variables, including older age, HCV viral load <800K IU/ML, FIB-4 score <1.45, absence of depression, diabetes, substance abuse, and use of DAA regimens without ribavirin were significant predictors of achieving SVR12. However, even in patients with characteristics negatively associated with achieving SVR12, success rates were high compared to success rates prior to DAA regimen availability. Patients with fewer comorbidities, better liver health, and lower HCV viral loads at baseline were more likely to achieve treatment success. Our results were consistent with other real-world studies, supporting the use of HCV therapy in HIV/HCV coinfected patients.
